# The Internalization of Stigma and the Shaping of the Grief Experience for Peers Bereaved by a Drug-Related Death

**DOI:** 10.1177/00302228241275728

**Published:** 2024-08-19

**Authors:** Daniel O’Callaghan, Sharon Lambert

**Affiliations:** School of Applied Psychology, 8795 University College Cork, Cork, Ireland

**Keywords:** drug-related death, addiction, overdose, peer loss, complicated grief

## Abstract

People who use drugs form a significant part of the community who are impacted by drug-related deaths, but their stigmatized positioning in society yields implications for their access to support and the social recognition of their grief. This project explores how the internalization of drug-related stigmas shapes the grief experience for peers bereaved by a DRD. Six individuals who experienced the drug death of a peer during their own time in active addiction participated in semi-structured interviews, analyzed by interpretative phenomenological analysis. Three superordinate themes are reported in this paper: (i) Forged Connections; (ii) The Condemnation Script; and (iii) Nowhere Left to Turn. Participants reported grief responses such as survivor’s guilt, shame, and increased drug use against the wider social invalidation of their close peer bonds. This paper appeals for a more health-based approach to supporting people in active addiction that recognizes and validates their grief experiences.

## Introduction

Drug-related deaths (DRDs) are a global public health concern, and bereavement for those socially connected to people who use drugs frequently results in a complicated grief with significant social and health implications (Bottomley et al., 2022; Titlestad et al., 2021). DRDs are deaths that occur directly due to the intake of narcotics but may also be a result of drug-related violence, suicide, or infectious disease (HRB, 2019; Titlestad et al., 2019). These characteristics, and the societal stigmas associated with drug use, often lead DRD bereaved individuals to contend with shame, isolation, and a lack of social support while grieving (Bottomley et al., 2023; Lambert et al., 2021; O’Callaghan et al., 2022). In approximation, 10–15 people experience bereavement from a single drug death (Dyregrov & Selseng, 2022), with research identifying complex and disenfranchised grief reactions in several family, community, and service provision contexts (Lambert et al., 2021; Meen et al., 2024; O'Callaghan & Lambert, 2023; Titlestad et al., 2021). Total deaths are increasing worldwide, such as in Europe (EMCDDA, 2024) and North America (CDC, 2022 as cited by Reime et al., 2022), identifying a critical and prompt requirement for targeted bereavement intervention protocols.

Research efforts in Ireland (Lambert et al., 2021), Norway (Titlestad et al., 2022), Britain (Templeton et al., 2017) and the United States (Feigelman et al., 2020; Sterling et al., 2022) continue to highlight the profound and complex impact of DRDs for bereaved family members. Collectively, the literature underscores diverse pre-, peri-, and post-loss experiences, with psychological affect stemming from anticipatory grief (Feigelman et al., 2023), the negative characterization of their loved one (Dyregrov & Selseng, 2022), and feelings of self-blame, shame, and disenfranchisement (Lambert et al., 2021; Valentine et al., 2016). The socially constructed narratives and moral stigmas associated with drug use compound grief processes to the extent that families often grieve in isolation, with prolonged exposure to complicated grief as a significant risk-factor for long-term health disorders (Doka, 2020). Family members are at increased risk for the development of depressive symptoms, prolonged grief disorder, post-traumatic stress disorder, generalized anxiety disorder (Bottomley et al., 2022), and a higher risk of natural cause mortality (Christiansen et al., 2020).

In accordance with Dyregrov and Selseng’s (2022) position that DRDs have a broad psychosocial impact radius, people in active addiction navigate a multi-disciplinary network of social, health, and justice systems. Consequently, healthcare professionals (HCPs) are also at risk for complicated grief outcomes (Sperandio et al., 2023; Yule et al., 2023). In contending with internalized expectations of professional durability, HCPs reported grief-related reactions inclusive of severe stress (Yule et al., 2023), guilt, self-blame (Sperandio et al., 2023), fears of litigation, anxiety, and traumatic flashbacks (O'Callaghan & Lambert, 2023). Negative social norms, sometimes integrated within organizational structures, heavily influenced the support available to HCPs, who reported difficulties with openly discussing the impact of DRDs (O’Callaghan & Lambert, 2023; Yule et al., 2023). Barriers to help-seeking can be further compounded by a complex amalgamation of internalized self-stigma and their societal positioning as experts in their profession (O'Callaghan & Lambert, 2023).

Evidently, the inhibiting properties of DRD stigma extend to the wider social context of drug use, and HCPs appealed for broader societal acknowledgement that people in active addiction form a vital constituent of the community directly impacted by DRDs (O’Callaghan & Lambert, 2023). People may witness multiple overdoses when in active addiction (Nolte et al., 2023) and, thus, are frequently positioned as first responders to overdoses among peers (Schneider et al., 2021). Overdose trauma has been associated with PTSD symptoms and, anecdotally, participants within the literature base reported that overdose deaths are increasing (Nolte et al., 2023; Schneider et al., 2021). Nolte et al. (2023) surveyed people who use drugs in New England (*n* = 578), where 83.6% of the sample had witnessed an overdose, and 85.8% knew someone who had died by overdose within the previous 6 months. In Toronto, Kenny et al. (2022) surveyed 249 people who inject drugs, and in the 6 months prior to participation, 79.5% witnessed 2 or more overdoses, and 41.4% witnessed 2 or more deaths.

Few studies have investigated the grief experiences of people who use drugs, yet the existing evidence highlights the prevalence of complicated grief reactions. Wojtkowiak et al. (2019) interviewed 10 bereaved men who use drugs, using grounded theory to explore their meaning systems within the social context of drug use. The participants’ stigmatized societal position inhibited their grieving opportunities, with many describing shared social spaces of meaning making, such as funerals, as “uncomfortable.” These experiences align with findings by O’Callaghan & Lambert (2022), who reported that some clients were forbidden from attending funerals. Denied of their right to engage in normative socioemotional grieving practices, social exclusion fostered a disenfranchised grief for participants, sometimes leading to increased drug use as a coping strategy (Wojtkowiak et al., 2019). This study presents a crucial insight into the lived experience of a critically under-researched population, but the exclusive focus on male participants may overlook potential gender-related variations.

Selseng and colleagues interviewed 13 drug death bereaved individuals to explore bereavement in the context of their own drug use (Selseng et al., 2023), and their experiences of support (Selseng et al., 2024). Both studies highlight the prevalence of complicated grief and a profound lack of social and professional support. Prompt and actionable supports are crucial (Selseng et al., 2024), as many participants reported using drugs to emotionally suppress their grief and avoid actualizing the loss (Selseng et al., 2023). However, while seven of participants in these studies were using drugs at the time of death (Selseng et al., 2024), others were not actively using drugs or were in treatment. Some participants reported returning to drug use post-bereavement, highlighting implications for recovery while underscoring a requirement for a nuanced understanding of the varying stages of drug use at the time of death. The importance of understanding grief at different stages is reinforced by Bethune Scroggs et al. (2022), who suggest that the bereaved may not begin to process losses until entering recovery; the death potentially including secondary losses such as companionship or safety.

Macmadu et al. (2022) utilized thematic analysis to characterize drug use behaviors in people following the overdose of a peer, identifying grief-related symptoms associated with fatal overdoses and increased overdose risks particularly evident when losing someone emotionally close to them (Macmadu et al., 2022). While this study included participants referring to both fatal and non-fatal overdoses, the work reinforces the scarce evidence base by highlighting grief-related symptoms in people who experience the fatal overdose of a social network member. The authors recommend future research to develop a more robust understanding, and identification, of mediators of these outcomes, including the effect of peer overdoses on individual overdose risk (Macmadu et al., 2022).

The lack of support for the bereaved reported by Selseng et al. (2024) may be further augmented by their limited social capital (Neale & Brown, 2016), with grief symptoms exacerbated by structural and socioeconomic inequalities such as poverty and criminalization (Petterson et al., 2019). Legislative policies may yield inadvertent consequences as they reinforce dominant social norms that devalue the bereaved person’s position in society, leading to poor health outcomes attributable to diminished help-seeking behaviors (Muncan et al., 2020). Thus, people who use drugs experience a double stigma, their experience of a stigmatized loss integrating with their socially stigmatized position as people who engage in behaviors perceived as unlawful (Selseng et al., 2023).

The current study aims to provide a phenomenologically rich exploration of how the internalization of drug-related stigma shapes the grief experience for peers bereaved by a DRD while in active addiction. Although scarce, existing literature highlights unique challenges faced by this population in being recognized as bereaved (Selseng et al., 2023; Wojtkowiak et al., 2019), and the current paper seeks to contribute to the establishment of a more robust evidence base for a significantly under-researched population. Building on previous research that focused solely on men (Wojtkowiak et al., 2019) or included people not actively using drugs (Selseng et al., 2023, 2024), this study specifically addresses people in active addiction, a subgroup highlighted for their grief-related reactions by Selseng et al. (2023, 2024). Addressing how stigma affects grieving processes in the context of ongoing addiction, this study aims to provide a holistic understanding of personally nuanced and complex lived experiences, offering a deeper insight into the intersectionality of grief, addiction, and stigma. Overall, it seeks to inform the development of culturally competent practice frameworks that are responsive to the influence of individual circumstances and experiences of trauma.

## Methods

The current study adopts a phenomenological approach to exploring the experiential impact of DRD bereavement for 6 individuals who were bereaved while in active addiction, utilizing interpretative phenomenological analysis (IPA) to present idiographic narratives and convergent themes across cases.

### Methodological Approach

The suitability of IPA as the chosen methodology is reflected in its hermeneutic capacity to explore individualized lived experiences, eliciting convergent perspectives which construct patterns of meaning across the sample (Bush et al., 2019). Emotional experiences of drug related bereavement carried a deep significance in the participants’ lives, with peer loss representing a major transition within the stigmatized sociocultural context of their addiction. IPA provided a phenomenological lens through which the psychosocial variability within the group could be interpreted, exploring how grief takes on a significance within the wider experience of active addiction (Larkin et al., 2021; Smith et al., 2009).

### Participants and Sampling

This study includes 6 participants (4 men and 2 women) who are now in recovery but were previously in active addiction and engaged with drug-related healthcare services. Utilizing purposive sampling, the authors sent emails containing information sheets to relevant organizations and other contacts within established professional networks. The email introduced the nature of the study and outlined the inclusion criteria, and the organizational leads circulated the information. Thus, participants could self-select to contact the first author by email or phone at their convenience.

After an initial distribution of the recruitment criteria that specified participants should be in recovery for at least 2 years, several people in recovery approached the first author expressing their desire to participate and share their stories. However, they did not fall within the specified timeframe for recovery. Thus, to minimize exclusionary criteria while ensuring emotional safety, experts in the field of addiction were subsequently consulted to determine a definition for recovery. These consultants reported that recovery is self-defined, wherein participants would have the right to self-determine. Thus, the inclusion criteria were finalized as follows: participants must have been over the age of 18, to have experienced the loss of a peer through a DRD during their own active addiction, to have completed a treatment program, and now be in recovery. Participants were also required to be actively linked with a support group, with access to support structures post-interview if necessary. There were no specific sex or gender requirements. However, efforts were made to secure an equal gender distribution in the sample where possible. Table 1 lists the pseudonymized name, gender identification, and the duration each participant self-determined their recovery at the time of interview:

**Table 1.** Profile of Participants.

Participant	Gender	Time in recovery
Levi	Male	8 years
Kenny	Male	6 months
Marco	Male	1 year
Annie	Female	6 months
John	Male	10 years
Sasha	Female	13 years

### Data Collection

The first author conducted 6 one-to-one semi-structured interviews between December 2022-June 2023. All interviews occurred in person, their duration ranging from 40-65 minutes, with an average of 46 minutes. Interviews adopted a semi-structured format via six open-ended questions designed to facilitate a participant-directed conversation about the experience of loss while in active addiction. Questions focused on their journey through addiction, the dynamics of their social relationships during this period, their experience with loss, the effects of stigma, and their sense of self. Participants were briefed at the beginning of each interview and debriefed at its conclusion, including the signposting of relevant supports.

Interviews were recorded by an audio device, from which the data file was transferred to an encrypted drive. Once each interview was transcribed verbatim with all identifying information removed, the original audio file was permanently deleted.

### Data Analysis

Data analyses utilized Smith and colleagues’ (2009; Larkin et al., 2021) case-within-theme approach to interpretative phenomenological analysis (IPA), with findings outlined in adherence to the standards for reporting qualitative research as reported by O’Brien et al. (2014). Both authors are proficient in working with qualitative data that details the lived experience of socially excluded populations, and IPA presents as a double hermeneutic yet systematic approach to deconstructing the experiential transcripts and synthesizing meaning within cases and across the dataset (Smith et al., 2009).

The first step involved familiarization with the data, where the transcripts were read and re-read. This was followed by the development of initial non-hierarchical notes, with an exploratory focus on explicit meaning and semantic content. A subsequent mapping of these notes by their interrelated concepts resulted in emergent themes (Smith et al., 2009). In this process, some notes were merged or discarded in thoughtful consideration about how to reduce the size of the data while preserving its complexity. Lastly, in searching for connections across emergent themes, the authors made decisions about how to contextualize the data within each case, resulting in the development of superordinate themes (Smith et al., 2009). In repeating this rigorous analytic process for each transcript, a cross-case analysis produced superordinate themes of higher-order concepts that best represented participants’ experiences of drug-related peer loss (Larkin et al., 2021; Smith et al., 2009).

### Roles, Reflexivity & Research Integrity

To faithfully represent the participants who kindly shared their stories for this project, the authors wished to ensure the highest methodological integrity. The first author has prior experience working with socially excluded populations through research-based practice, and the second author has both practitioner and research experience in addiction services. As the sole interviewer, the first author wrote post-interview memos and engaged in reflective journaling to diarize an audit trail from the beginning of the analytical process. These notes were generated to facilitate transparent discussions in peer debriefing sessions between the authors. Debriefing sessions adopted a reflexive bracketing framework (Smith et al., 2009), with consideration for emerging feelings, potential biases, and pre-existing assumptions. Addressing the risk of bias through reflective practice and peer debriefing established an iterative dialogue that coincided with our responsibilities as qualitative researchers (Dodgson, 2019).

### Ethical Considerations

This study’s design was not preregistered, and thus received entire ethical approval from the authors’ home university through its social research ethics committee. All pre-interview materials ensured participants were fully informed of the study’s nature, the details of participation, and the potential uses of their data. Participants provided informed consent via signed consent forms before their interview. Any identifiable data was anonymized and confidential, and participants were informed of their right to withdraw their materials from the study up to 2 weeks post-interview. Upon completion of the anonymized transcripts, the original interview recording was deleted, and data was stored in written format (Microsoft Word) on an encrypted Microsoft OneDrive provided by the authors’ home institution.

## Results

Presented herein are three superordinate themes (Table 2) demonstrated through a case-in-theme approach to idiographic narratives: Forged Connections; The Condemnation Script; and Nowhere Left to Turn. Each superordinate theme contains three subthemes, which categorize narratives of particular focus under the broader superordinate theme. Collectively, these themes illustrate complex journeys of grief as participants navigated the loss of companionship amidst social invalidation of their peer bonds. Through discriminatory societal practices and belief systems that frame addiction as a criminal activity, grief-related reactions were inarguably shaped by how addiction is situated as a taboo within the social domain.

**Table 2.** Superordinate Themes With Descriptive Context and Associated Subthemes.

Superordinate theme	Subthemes	Description
Theme 1: Forged connections	• Filling a void	Explores how grief takes on a particular significance in the context of the intensity of peer relationships, where the shared experience of social exclusion enhances the peer bond.
	• Social resonance	
	• Cost of belonging	
Theme 2: The condemnation script	• Shattered reflection	Illustrates how participants conceptualize their grief, disenfranchised by stigmatized societal belief systems that criminalize addiction and invalidate their grief.
	• Social fracture	
	• Inhibited grief	
Theme 3: Nowhere left to turn	• Drug dependency	Demonstrates how participants respond to their grief in contention with stigma while bereft of social support.
	• Death as an escape	
	• Survivor’s guilt	

All participants spoke solemnly about multiple peer losses throughout interviews, but each referenced close friends whose deaths were significantly impactful for them. To provide additional context to the individual lived experience of each participant, Table 3 contains a brief overview of these relationships and the circumstances of their deaths.

**Table 3.** Participants’ Relationships to Deceased and Circumstances of Drug-Related Death.

Participant	Social context of death
Levi	Levi’s best friend and godfather to Levi’s child.
Kenny	Close friend died by overdose and was found in a public bathroom by authorities.
Marco	Childhood best friend, Cody, who died while both were using together in Cody’s home.
Annie	Close friend who Annie had met during an early treatment effort died when both left the treatment center prematurely.
John	Best friend Ben, whose drug-related death caused significant pain as John still grieves his potential.
Sasha	Two closest friends, Henry and Ava, who died 3 weeks apart.

### Forged Connections

Societally ingrained stereotypes about participants’ place in the world created social barriers between themselves and the wider community. Accordingly, they constructed their social world within the margins that they felt socially relegated to. Adopting and internalizing dominant social perceptions, addiction became a defining feature of their identity, pushing participants further to the periphery of society and increasing their sense of isolation. As poignantly stated by John, the public fails to recognize just how pervasive such deeply prejudicial attitudes can be, severing the connection between people who use drugs and the wider community: *“When you’re using [drugs], you feel every bit of [the stigma]. So, you just feel like you’re outside of society and you’re eh… You know, it’s just very isolating.”* For all participants, however, existing on the fringes of society forged connections with others who were similarly alienated, serving as an acute juxtaposition to the hugely isolating nature of addiction. As highlighted by Sasha, the shared trauma histories and social exclusion pushed peers closer together, carving out an interdependent, non-judgmental social space:

I just feel that those relationships at the time, they did form something, they did provide some form of connection. And when you're in a lonely place like addiction, you know... Any connection is better than none.

An often-unspoken interdependency, the shared nature of their socially assigned position on the outskirts of society constructed bonds that kept peers alive during severely chaotic life situations. As a result, drug-related stigmas yield the dual-outcome of exacerbating the social isolation of people in active addiction while concurrently enhancing peer bonds within this community. The critical role these connections played in the lives of participants, bolstered by stigma, often resulted in intensified grief reactions when a peer passed away.

#### Filling a Void

Many family figures in Levi’s life were unable to fulfill designated roles, and he positioned his peers as surrogate role models in place of absent caregivers: *“I was diagnosed with all sorts of stuff from a very young age. Dad was in and out of jail, so I turned to the streets for that friendship. For that male role model.”* As his peers came to personify these roles in his life, Levi described a sense of kinship and familial bonding among a *“community of people”* that were situated on the margins of society; a conceptualization of peer bonding that was reflected in the narratives of all participants. The concept of ‘family’ was a value of central importance for Levi who, in demonstrating how his personal values augmented the importance of his peer bonds, shared that many of his peers acted as a proxy for his biological family: *“The friendships I formed were basically my family…I knew then, I wasn’t the only one in this situation*.” In a similar manner, communal acceptance acted as a core cultural value for Kenny, and the disempowering effects of social exclusion were magnified due to how his personal self-worth was intrinsically linked to how he was perceived by those around him: *“For me, I always felt that I was ‘less than’…and I had that even before I started taking drugs.”* However, Kenny’s peers in active addiction afforded him a sense of belonging he had yearned for, forging bonds that replenished his sense of place with an implicit sense of acceptance:

It’s a cutthroat lifestyle. You have to be street wise, and you have to watch your back but there is kind of a community there as well, like you know? I think it’s like, “we all know we’re f**ked.” But we’re okay with each other like, we’re okay with each other…

Levi and Kenny’s narratives demonstrate how such relationships can become deeply meaningful as they represent the personal fulfilment of human connection that social exclusion deprived them of. Comparably, friendship and connection epitomized the core of Marco’s character, and despite underlying insecurities about his self-worth, his morale was largely fortified by his attachment to his childhood best friend: *“My best friend Cody, he felt like a brother to me. Basically, I would have taken a bullet for him. We’d travel the world together.”* In contrast to other participants, Marco’s relationship with Cody existed before engaging with drugs, but drug use ultimately became a distinctive feature of their brotherhood as they traversed the threshold from recreational drug use into active addiction.

As the essence of Marco and Cody’s connection changed, the value remained. Marco cherished this friendship, with his reliance on their bond becoming more intense due to increasing levels of social isolation stemming from their drug use. This bond acted as an anchor to Marco’s sense of psychological safety, demonstrated by how Cody’s death took on such significance in Marco’s world as it began a sequential process of loss: “You feel invincible, bulletproof. Little did I know I was made of f**king eggshells… Lost my job, banned off the road, apartment gone. I literally went off the edge.” While Marco’s narrative highlights how existing relationships can change within the bounds of addiction, it further demonstrates how stigma and isolation can forge powerful and meaningful bonds among peers.

Levi longed for the wider societal recognition of these connections as authentic and meaningful; that the terms ‘family’ and ‘friend’ carry the same merit regardless of your social situation. Levi’s best friend died from a DRD, and he shared how he honored this man’s humanity both in life and death: *“I've a tattoo on my arm of him, he’s the godfather to my child. I didn’t make him that, or get tattooed, if it wasn’t a significant relationship in my life.”* While many of these relationships were predicated on a need’s basis, particularly around drug distribution, such principles did not negate the development of meaningful friendships.

#### Social Resonance

The community of people who use drugs are regularly impacted by cycles of death, incarceration, and grief. The constancy of change emerging from the cyclical loop of trauma and crisis impedes opportunities to process and make sense of these experiences, but one constant for participants was the homogeneity of shared experience which often brought people closer together. Shared experience acted as a synergetic social mechanism by which the depth of peer connections authenticated, and within these friendships, participants were identified by the qualities of their person and not by the characteristics of addiction. Sasha found solace in the escape that drug use provided as a shared experience amongst peers and noted that deeply traumatic circumstances engendered a collective emotional understanding: *“There’s a lot of emotion around the pain we share. I look back on those times and I see those friendships as inevitable…If there is no safe adult, peers will lead peers.”*

Mutual understanding was particularly pertinent for Annie, whose close social contacts failed to understand that her addiction was a health issue, perceiving her as making poor ‘lifestyle choices’ and influencing her social and familial detachment. Among her peers, including those within various treatment centers, Annie felt *“seen”* in a way that contrasted how she felt socially imperceptible by wider society. She acknowledged that an unspoken communal essence among peers eventually manifested into the formation of strong connections, cultivating the sense of belonging and understanding that Annie had lost:

All those girls, I’m still in contact with a good few of them. There's just such a connection there, you know? Because you’re so lost and you’re so alone when you go into a treatment center, and finally you have somebody that is experiencing the exact same things you’re experiencing.

Participants did note that peer groups in this context were not in the best position to be supporting one another, but as they were marginalized by society and in deep crisis, the concepts of shared experience and mutual understanding played a significant role in buffering against the stigma from an intra-community perspective. With drug use interwoven into the fabric of their attachment, Levi acknowledged an underlying transactional element to these relationships, but he inherently sought to satisfy the intrinsic human desire for connection. Thus, shared experiences created solidarity amongst disenfranchised peers:

[We had] really close bonded friendships, even though it was around substances. But [my peers] were all coming from traumatic experiences as well. And they were on the streets too, so who better to support one another? You know, through substances, but also through friendship.

Sasha echoed these sentiments, acknowledging that many of these relationships were predicated on behaviors, actions, and principles that can be considered dangerous, but they bonded through the desire for belonging and the underlying sense of mutual understanding:

[The connections] weren’t necessarily healthy, but people will peer up because we're social animals. And we gravitate, we're wired for connection, even if those connections are toxic…So my peers at the time would have been all young people like me who were equally strung out and distressed for multiple reasons. And I would have had a couple of close friends... Everybody was dead by the time I hit 25.

#### Cost of Belonging

Such belongingness and intense peer bonds were costly, and the mortality risks of active addiction presented a perpetual threat to this socially constructed safety net. With stigma concealing their support needs from mainstream society, participants were left to grieve significant losses as they remained encircled within their own temporality without external intervention. Cycles of loss depicted the fragility in which each participant’s sense of belonging was predicated on, and Kenny noted the challenges of dealing with the death of a peer that represented so much during a time of ongoing crisis: “*There is a community. The people do kind of look out for each other. It is community and a death does hit people as well, and it does hit people who are, everyone’s in crisis as well.”* As John stated, the lack of societal acknowledgement of the rupture caused by DRDs means that the grief and impact is often contained within the community “*the community is left with it.”*

John spoke fondly of a close friend Ben, and to him, their closeness was as profound as any relationship in his life. However, when Ben died, he experienced a profound grief that he was left to contend with in isolation. John deeply connected with the loss of life and the loss of potential, grieving the humanity, kindness, character, and potential of Ben that would never reach beyond the limits of his social status:

He was a very close friend now. He was very funny as well. And he was a great singer. And he's from a nice family as well, he was good natured...When I think of when we do functions and social gatherings, stuff like that, I think of what Ben is missing out on.

The concept of ‘potential’ embodied a recurring thread within these narratives of grief. Participants paid respect to the peers they had lost by conceptualizing their lives in terms of their humanity, remembering the human qualities which they never had the opportunity to actualize in wider society. Participants learned that such connections came at a high price; they provided psychological warmth during a time of crisis, which then resulted in a profound grief when a peer died by drug-related circumstances. Annie analogized such grief to the loss of a family member: *“You’re just so close to those people and I… I mean I grieved now like it was a parent.”* Drug-related peer loss detached the anchor of belongingness that Annie had fervently clenched, and she longed for the recognition that such connections carry authenticity.

Marco cited the loss of Cody as the driving force for what *“really got [him] on heroin,”* accelerating his drug dependence and shattering his worldview as he transitioned from smoking to intravenous dependency. The psychosocial implications of the loss were amplified by the traumatic conditions in which it occurred, as Marco was not only forced to contend with the loss of his best friend, being the first to discern that he was in difficulty, but furthermore, to bear witness to the ensuing panic:

We were smoking in his house, and we goofed off, could have been hours, six or seven in the morning at this stage. I woke up, and he was blue on the floor. After not breathing. I tried to give him CPR, his dad came up then and was like “he’s fu**ing dead!” I was ringing the ambulance, they came and had the machine over him, a chest pumping machine and all his ribs were breaking. This was unbelievably traumatic. Because he was my best friend, and he was dead next to me in the bed.

### The Condemnation Script

Drug-related peer deaths represented the loss of a key sustaining factor that provided solace from the pervading sense of loneliness that participants experienced while pushed to society’s margins, but also the loss of familial connection, friendship, and human potential. Articulated by Levi, outside of their community participants witnessed their peers reduced to dehumanized statistics in death, fostering a sense of futility about their own existence: “*It’s not acknowledged or they’re ‘just another one’...We’re just another statistic.”* As peer deaths caused a significant rupture in the social world of participants, this was not the case at a broader societal level. The divergence between the personal and societal reactions to the death created a psychological incongruency for participants whose meaning-making processes were subsequently impeded by the social nullification of their peer bonds.

The invalidation of these relationships caused harmful introspection about the value of participants’ own lives and affected their ability to sustain other relationships. They were condemned for their association with drug use before they were recognized as human beings grieving their loved ones. Furthermore, all participants were disheartened to witness the same labels their peers had grappled with in life remain attached to them in death, prompting a reflection on the value of their own existence. As stated by Kenny, such condemnation perpetuated a fear that their own lives were meaningless, with labels indoctrinating the false notion that one’s life can carry less meaning based on social currency:

That was a big fear of mine then. That I’d be found dead in a toilet and labelled as a junkie. I’m a person too. No one wants to be like that, it’s a brutal way to die as well.

#### Shattered Reflection

The psychological dissonance following the death of a close friend presented significant challenges for participants, as the value they placed on the lives of their peers ran contrary to society’s incognizance of the loss of human life. Given the significance of shared experience, participants often saw themselves reflected in the lives, and deaths, of their peers. Contradictory belief systems convoluted their efforts to make sense of the loss, particularly as it instigated introspective processes of self-evaluation. Stigmatized reactions to the deaths forced them to contend with the double stigma of grieving a societally condemned death while also attributing these social attitudes to their own person. The ingrained belief system that drug use devalues an individual’s place in the world was reflected in Annie’s immediate familial context with little consideration for how this may prompt harmful introspection:

I see it in my own family. If I tell them someone has died [by natural causes], or someone has died from a drug related death their reaction is so different. It kills me. And sometimes I don’t even talk about it. Because I’d get so angry, I’d internalize it and I’d feel it inside. I’d be frustrated, and so angry with them for their reaction to it. So, I don’t talk about it.

Internalizing these reactions led to a marred self-concept, but also inhibited Annie’s help seeking behaviors due to substantiated fears that her grief would not be taken seriously. Furthermore, normative grief-related reactions such as anger and sadness intensified as they emerged from both the grief itself and the indifference of those around her. When grieving, Annie was starkly reminded that despite the love she held for the deceased, such compassion was not universally conveyed. Through tainted self-reflection, Annie made sense of the loss in the context of her own drug use as she became concerned that her own death might bring shame to her family amidst a backdrop of societal apathy:

I kind of worried, am I going to die from, is it going to be a drug-related death? Will it be shameful on the family, will people even care as much because it’s a drug related death and not natural cause? And it’s mad that I’m even thinking about, ‘how am I gonna die?’

Kenny experienced similar challenges about his place in the world, consequently questioning the value of his own life. With no symbolic representation for a life outside of addiction, he acknowledged his grief through sorrowful compliance that his societal footprint would be minimal, shrouded by the socially defined undesirable characteristics of drug use. Seeing himself reflected in the faces of those who died, each death set in motion an internal grieving process as he questioned if he would be mourned:

I knew there was a potential that I could die any time and I just accepted it like, you know? Would anyone care? Part of you, then, wants to die, man. Part of you is okay with dying. Life is so s**t anyway.

The internalization of dominant social norms stripped Kenny of both his humanity and individuality. Social condemnation and societal disregard for the death of their peers further diminished self-worth for all participants, as their already diminished sense of self was shattered by the perception of their peer community as meaningless. John spoke at length about his own self-worth during this time, his sense of self eroded as he internalized the socially perceived traits of the community as individual characteristics. However, he implored society to recognize that each person in this community is left with a complex and individualized grief: “There's a lot of grief left through the people that are using [drugs] and they’re not f**ing robots; you know what I mean?”

This deindividuation resulted in the formation of a dual identity for participants, where the human beyond the addiction was not socially acknowledged. When John’s best friend Ben died, society failed to align the dual identities that John carried, and in the wake of societal inattention towards his bereavement, his personal efforts to align both identities became more challenging. Ben was an individual who had boundless potential, and in grieving the loss of his close friend he also grieved for his future and his humanity. Despite John recognizing his potential, Ben could not detach from the stigmatizing labels imposed on him, resulting in self-questioning about what this suggests about his own prospects:

What he would have brought to life, and the potential he had, because when somebody dies... Not Ben the 30-year-old heroin addict. It’s not just him that died. It’s the father he could have been, it’s the psychologist he could have been, or the care worker, or whatever he wanted to do with his life. The contribution he could have made to society.

#### Social Fracture

The indifference of society towards the shared grief of the community impacted many existing relationships that participants held, exacerbating their sense of isolation following a death. Sasha recounted a lack of support, struggling to receive any help at all, so much so it impacted on her existing relationships. Following the deaths of her two close friends, Henry and Ava, Sasha’s health deteriorated, and she retreated from her remaining social circles who did not recognize the depth of her grief. However, the attitudes by those around her, even those who would be able to provide support, exacerbated her grief:

After that, I hit rock bottom. And at this point no doctor would touch me. It was just “look, she's going to die anyway,” d’you know? ‘Cause I was just so, so using at that point. There was grief at the time, but then I realized how much trouble I was in myself and I kind of shut down around the relationships I had developed.

For Marco, grief-related reactions by those around him impelled Marco to internalize the perception he was complicit in Cody’s death and permeating his own grief with socially reinforced self-blame. Marco recounted his desperation as he made vehement efforts to wake Cody and save his life, his best friend unresponsive in his arms as distress reverberated throughout the household. His efforts to revive him fostered feelings of intense futility and self-blame which were exacerbated by Marco’s vilification in the aftermath, his friendship with Cody now socially recognized as a blameworthy causal factor in his untimely death:

There was an inquest. When I went down, I felt like I was being attacked. All his family were after coming, and they were like “what the f**k...” Obviously, they were channeling their anger, their son was after dying and I understand that now. But at the time I was like “what the f**k, I’m his best friend, I wouldn’t do anything to hurt him.”

As vilification from people connected to Cody augmented Marco’s sense of failure and culpability, descent into such a belief system fueled his crisis of self as he subscribed to the epithets imposed on him: “*It was the absolute worst…All the demons came back tenfold, telling me I’m useless...Waster, a failure your whole life. Heroin was a big escape for me, and tablets.”* In his efforts to find meaning in the loss of a cherished friendship, the intersectionality of complicated grief, existing traumas, and condemnation trapped him in perpetual rumination over his perceived role in the death. Their friendship transcended addiction, but the qualities of Marco and Cody’s relationship were disregarded as addiction became its key identity marker. Drug use altered the social perception of their bond and overrode Marco’s permission to grieve. After the breakdown of his connection to Cody’s family, in reflecting on how they were forced to contend with their own stigma Marco recognized that he now embodied the circumstances of their son’s death: *“He [Cody’s father] probably still blames me to this day; I’d never spoken to him again and I used to be up with that family every Christmas.”*

#### Inhibited Grief

The psychological retention of stigmatized labels impeded all participant’s engagement with culturally normative grieving rituals, and these challenges manifested differently depending on circumstances personal to each participant. Layered with underlying stigma and given his positioning as complicit in his peer’s death, Marco’s interactions with others throughout the grieving process acted as further inhibitors to engagement with grieving rituals. Drug-related stigma and blame socially resituated him from ‘brother’ to ‘unwelcome guest’ at the funeral: “*You get those eyes watching you like, ‘oh he was with him when he died.’”*

Annie’s relationship with her family also became strained throughout the grieving process. In losing a close friend to drug use, Annie’s expression of normative cultural grieving practices suddenly took on a new meaning due to stigmatization associated with drug use. Publicly grieving the loss of her peer became a subject of question as the validity of her connection was disregarded, contributing further to Annie’s sense of disenfranchisement:

I watched it online, the funeral. Jesus, I was just heartbroken. My family then looked at me saying “she hardly even knew her,” you know? “She was only in treatment with her.” But that connection you make with people in a treatment center, it’s just unbelievable.

Kenny’s engagement with culturally normative grieving rituals such as funerals, as routine social practices to honor his friend’s life became veiled in uncertainty and apprehension: *“You don’t fit in…I tried to pay my respects, though I find it hard going to funerals. I feel less than, I don’t even know if the family wants me there. I still went because he was a friend!”* Such feelings of apprehension were reflected in the narratives of all participants; John reported similar psychological difficulties when attending funerals: *“I remember going to the funeral. I didn't want to go. I just barely went to show my face and legged it again… Just felt really awkward about it, you know?”* Participants’ inability to comfortably pay their respects due their association with drugs severed their sense of belonging, further contributing to their sense of disenfranchisement as drug-related stigmas obstructed their perceived right to grieve meaningful relationships. However, their proclivity to attend funerals despite prejudice and while actively in crisis illustrates the high regard to which participants hold these connections.

### Nowhere Left to Turn

The reactions of others to the deaths of their peers confirmed participants’ fears that the wider public considered their lives and deaths as inconsequential. Left to cope with complicated grief on their own while their sense of belonging dissipated, this gradually bore significant repercussions for their social network, adversely affecting their intrinsic sense of community and access to support. With limited options for support, Levi described his social world as *“absolute chaos”* following the death of his best friend as the bereavement inflated an excessive emotional load already saturated by a myriad of social, justice, and health complications:

You had all the life situations as well that were going down, with social workers, kids, courts. Family members, dependency, money. All that stuff that you couldn't deal with anyway. So, then you throw this loss on top of it. You're just scattered.

The overwhelming combination of grief, trauma, and fear with no social support to assist in the process of overcoming this immense suffering left participants with little option but to remain trapped in cycles of suppression. Sasha recounted similarly chaotic experiences as she could not regulate around the death of her friends: *“We learned to shove down grief and just keep surviving ourselves.”* With depleted capacities for emotional regulation and no external intervention, herself and her peers were forced to rely on each other as they had nowhere left to turn. However, as most members of the community had inhibited coping skills, they were often not in the best position to support one another. Their community was in crisis and existence within this domain exacerbated her sense of grief and trauma:

Suicide attempts, overdoses. It was a lot of chaos, and there was very genuine reasons for all of it. And the help certainly wasn't there. But I think in our peer mix we just could not help one another. Because we were all in that boat. And I think our efforts to help one another were probably more harmful and more toxic.

Society could not recognize the psychological turmoil that Sasha and her peers were experiencing. She highlighted the complexity of emotions that, upon reflection and therapeutic intervention, are now discernable but created a cascade of disarray during a time of crisis:

Yeah, drug use increased. I think when Henry died, I would have been particularly close to him and I had just been to Ava’s funeral 3 weeks earlier, and we were all kind of in a circle...We were very close. I just felt at the time, there was guilt. There was shame. There was a lot of things. Fear. And definitely, I suppose, in lucid moments, just a reflection on how we were living our lives, but also a deep frustration about not knowing why the circle was continuing and nobody was asking ‘why,’ or looking to help.

#### Drug Dependency

With a limited emotional bandwidth to engage in loss-oriented practices, and a scarcity of professional resources to assist with psychological restoration, participants’ existing drug dependency continued to function as their core coping mechanism. Overwhelmed by grief, the duality of mourning a stigmatized loss in a stigmatized community initiated a causal sequence of increased drug use, where all six participants reported a rise in drug intake following peer loss. For John, with a lack of targeted supports and his life socially governed by stigma, the grief compelled him to rely on drug use as a numbing agent:

Because I didn't want to feel what I was feeling. It didn't scare me away from using drugs. In fact, it just spurred me on to use more. Because the grief, the upset, the hurt, the pain. You don't want to feel that, and you just want to take more tablets.

Following the DRD of a peer, Kenny’s fear center became considerably over-activated as he faced the reality of grief and, subconsciously, what that loss represented for his own mortality. Entrapped within the boundaries of social stigma with little options for support, he entered a phase of psychological paralysis, compelled to draw on his existing coping strategies to quell the emotional noise in his head:

It could happen to me tomorrow... It didn’t stop me using [drugs]... If anything, I’d have used more. Just to kind of push down the feelings, and now that you’re in danger yourself. It’s a reality, but if you’re using [drugs], you’re not worrying about it.

Increased levels of drug use placed participants at a further risk of fatal overdose, and this grief response involved the very behavior that participants were socially condemned for. Levi acknowledged that masking the grief with substances may be societally misconstrued as indifference towards the loss of his close friend, however, this was not the case: *“They weren’t understanding the different situations that I was after going through. That led me to picking up that substance.”* The unseen reality was, as Levi articulated, that drugs acted as a suppressing agent for participants as there was no capacity to manage more emotional pain:

I didn't know how to manage [the death], and the only way I knew how, by every other situation in my life, was to use drugs. So, the drug use increased. Because I just wanted to numb all of the feelings. I didn't want to feel anything. And that's what drugs do.

The presence of drugs as a causal circumstance in Cody’s death had an inverse effect on Marco’s opportunities to grieve and isolated him in the aftermath. Internalized feelings of blame and shame through stigmatized vilification negated his propensity to engage in help-seeking behaviors as his drug use escalated. A relationship that had once provided him psychological warmth shifted to a source of psychological trauma; the conceptual reorientation of a cherished bond resulting in increased drug use and long-term psychological outcomes:

I was diagnosed with PTSD from that, from all the stigma. When I was at the inquest the dad was shouting and roaring at me...Heroin and tablets went just through the roof. I mean, bad. It was just downward, a proper “forget everything” type of thing. 30 Xanax a day, and a bag of heroin. I’m lucky to be alive like, I swear to God.

#### Death as an Escape

The concatenation of events following the death ruptured Marco’s ideological interpretation of the world and he struggled to find meaning within the remnants of his social domain. Ultimately, a sense of hopelessness prevailed as Marco expressed his difficulties living in a world without a place for him. His world had become psychologically cold, and a very dark place: *“If anything, I wanted to be in [Cody’s] position. At that time, I wanted to die like. My whole world was after falling apart.”* Sasha disclosed similar expressions of envy, struggling to exist in a world where she felt so isolated and could not attribute meaning to her own life: *“I can almost remember being envious of young people that were part of my peer group because their pain was over, and I couldn’t understand.”* For Annie, the ceaseless condemnation of her peer group over years of social exclusion and apathy towards their deaths eroded her self-worth as she projected society’s indifference on to herself, she was envious of the fact that her peers were no longer suffering while she was left behind without a place to belong, and to endure continuous denunciation:

When I heard about somebody who died from drugs, I was jealous. Which is awful, but I wasn’t in a good place myself and I felt I was never going to get out of this, and I just wanted to die myself. And I was like “why can’t that happen to me?”.

Given what such meaningful connections represented for participants during times of severe crisis, such as kinship and mutual understanding, the impact of the deaths of individuals representing these intrinsic human values became far more complex. All participants spoke about their relationship with death during their time in active addiction, with Annie disclosing that, trapped in a cycle of trauma but longing for peace, she attempted death by suicide:

It started to impact on every area of my life. Friends, financial, and mental health was very, very bad…I was taking drugs quite a lot. It really did take over so I started stealing money from everywhere I could. I would try pay for things and everything, so my family started to find out. And work, everything. And so, then I tried to take my own life twice.

Annie’s drug use permeated multiple facets of her life as existing relationships became strained over time and some, including her marriage, deteriorated entirely. Expressions of suicidal ideation or attempts to die by suicide emerged throughout interviews as participants felt that they had nowhere else to turn. Kenny’s primary coping resources centered around repressing extant traumas, and the reinforced sense of disenfranchisement emerging from the bereavement necessitated more urgent coping strategies. However, Kenny was so emotionally overwhelmed that he could not permit himself to grieve, nor could he allocate the psychological resources to make sense of the loss in the context of broader society. As a result, he became enmeshed in a cycle of emotional suppression and suicidal ideation:

I made the decision to kill myself... I was very badly strung out, I was like, “so I’m stoned today, but tomorrow is coming. I’m going to have to come down sometime and I just can’t do any more sicknesses.” And I just was like “alright then, it’s okay, you’re going to kill yourself, you don’t have to do it anymore.”

#### Survivor’s Guilt

Many of the participants explicitly stated that, to this day, they live with survivor’s guilt that manifests in personally individualized ways. Marco, who felt blameworthy for Cody’s death and has not reconnected with Cody’s family, questioned the sanctity of his own life as he dwelled on his survivor’s guilt. Even in his recovery, the internalized shame of his past remains very much alive:

I was driving, his mother walked past me, and I put my head down. To hide. Because I was ashamed. Over that stigma being put into me, I used to use [drugs] with her son. And now he’s dead and I’m alive, and here I am driving a car. I just felt awkward and guilty, and I know this woman since I was 10 years old. That’s the madness of it.

John wished for more conscious efforts to recognize the potential and humanity of people in active addiction; to create visibility for life outside of addiction and to break the cycle of psychosocial entrapment within a continuum of trauma and social neglect. However, following Ben’s death, John experienced profound survivor’s guilt as he considered his own life in comparison to how he viewed Ben as a beacon of potential. Contending with survivor’s guilt alongside the social conceptualization of his grief as unsanctioned hindered his efforts to make sense of the loss:

I'm feeling like, “how the f*** did he die?” He's only using [drugs] about the last 12 months, and he's innocent. I'm using about the last 10 years, heavily, and I'm here and he's not, you know?

## Discussion

The current study shared the voices of six bereaved individuals who experienced a DRD while in active addiction, using IPA to center on their narratives and explore how their grief experience and identities as bereaved were shaped by stigma. For participants, their peer bonds represented mutual understanding, companionship, and familial connection, where drug-related peer loss resulted in profound grief-related reactions. Influenced by the twofold stigma of grieving a DRD while positioned within a stigmatized community, participants reported a diminished self-concept, survivor’s guilt, self-blame, shame, and increased drug use against the societal invalidation of their bereavement. The findings highlighted an urgency for targeted bereavement intervention protocols that acknowledge an increased risk of fatal overdose and disenfranchised grief for a community in crisis. The firsthand stories of grief portrayed by the bereaved in this paper corroborate healthcare professionals’ implorations that people who use drugs are critically underacknowledged as a group who are impacted by DRDs (O'Callaghan & Lambert, 2023).

### Discussion of Empirical Findings

This study further reinforces the conceptualization of drug-related bereavement as a complex area with no uniform approach to support. In providing an in-depth phenomenological insight into individual mediators of grief for people in active addiction, and the convergence of bereavement experiences across this group, this paper demonstrates that grief outcomes are influenced by a nuanced interplay between personal experience, systemic belief systems, and legislative structures. However, while the participants in this study reported many similar complexities in their grief responses to other social groups such as families (Lambert et al., 2021; Titlestad et al., 2019) and HCPs (O'Callaghan & Lambert, 2023; Sperandio et al., 2023), there are significant contextual differences. The narratives provided by participants provide a crucial insight into the complex mental processes involved in grieving a stigmatized loss while socially situated within a stigmatized community.

To this end, the current study supports the identification of a unique double stigma highlighted by the work of Selseng et al. (2023, 2024), and comprehensively explores the health implications of grieving while experiencing marginalization and compromised wellbeing. Most crucially, this study underscores the immediate aftermath of a peer’s DRD as a critical period for crisis intervention as participants expressed suicidal ideation, attempts to die by suicide, and increased drug use with a further risk of overdose. With systemic stigmas generating both internal and external barriers to help-seeking and support, the psychological integration of these experiences stretches beyond the individual capacity of those who are already in crisis. With difficulties regulating around the loss of their peers, the findings reinforce a growing evidence base that identifies increased drug use as a persistent grief response for people who use drugs (Macmadu et al., 2022; Selseng et al., 2023), necessitating urgent community-based strategic interventions to prevent further loss of life.

This paper advocates for drug-related policies and procedures in addiction, health, and homelessness that recognize and employ actionable responses to the increased risk of further deaths by overdose and/or suicide within communities after a DRD. Furthermore, this study provides in-depth contextual and relational information about barriers to help seeking through an exploration of socioemotional intricacies, which services can utilize to mitigate these barriers and increase the efficiency of crisis support provision. Broadly, participants noted that their grief was unrecognized by the wider public, echoing the findings by Selseng et al. (2023, 2024) who reported that, with their humanity and grief neglected, the bereaved remain solely positioned as people who use drugs and not as people who grieving a profound loss.

While participants acknowledged the risk factors of forming attachments while in crisis, this study provides additional in-depth context to the evidence base regarding the function and importance of peer relationships in active addiction. All six participants analogized many relationships to various roles within a family structure, with their peers representing social intimacy, belongingness, and potential, adding further credence to Bethune Scroggs and colleagues’ (2022) reporting of drug-related peer loss invoking secondary losses. Macmadu et al. (2022) noted that increased overdose risks were evident when the DRD of an emotionally close peer occurred, and in the current study, all six participants acknowledged the loss of at least one close relationship that had profound implications on their wellbeing. However, drug-related stigmas eroded the social validity of these peer bonds.

Their peer bonds delegitimized during life, and their grief disregarded during death, this report highlights a unique disenfranchisement for people who use drugs as their connection to the deceased is not formally defined. Although participants used heartfelt familial terms to describe their peers, the personal meaning of such connections may not be socially acknowledged in the same way as more universally recognized relationships, such as a parent’s connection to their child. Wojtkowiak and colleagues’ (2019) work demonstrated a fragmented and delayed grief for this population, and Bethune Scroggs et al. (2022) stated that the individual may not begin to fully process the loss until entering recovery. These findings are reflected in the current study, as participants struggled to find meaning in the loss of their peers, which had further psychological implications for their self-concept.

Given the prevalence of loss in the lived experience of each participant, and the broader context of active addiction, urgent action is required on behalf of addiction and recovery services to provide information on delayed or disenfranchised grief. Currently, there is a societal failure to look beyond addiction to recognize the individual who is grieving in crisis, and the professional acknowledgement of grief within services would serve to validate and support those who have been bereaved by a DRD, including recognizing ‘survivors' guilt’ experienced by peers. Furthermore, strengths-based therapeutic interventions that assist the bereaved in making sense of the loss are recommended, especially given the profound impact on participants’ self-concept reported in the findings.

Validating the peer bonds for this population may yield implications for posttraumatic growth as, amidst the stigma, both families (O’Callaghan et al., 2022) and HCPs (O'Callaghan & Lambert, 2023) identified role constancy as a pathway to healing. Parents maintaining continuing bonds with their children through symbolic means and HCPS utilizing their professional responsibilities as practical coping mechanisms functioned as both loss-oriented and restoration-oriented methods of healing (O’Callaghan et al., 2022; Stroebe & Schut, 2010). By internalizing the social undermining of their peer bonds, participants’ roles in the lives of their contemporaries became unclear and, in some cases, nullified. Coinciding with the findings of Wojtkowiak et al. (2019), opportunities to honor the lives of their peers were severely inhibited, as participants felt unwelcome at funerals and shared spaces of mourning evolved into areas of judgement and condemnation despite their efforts to pay respects.

Dominant social norms construct attributional beliefs at a societal level that frame drug use as a behavior that is morally wrong, neglecting to recognize addiction’s association with trauma. These norms, reinforced by inequitable societal and legislative structures, can perpetuate stigma and hinder support practices through a justice led reaction to a mental health problem (Muncan et al., 2020). Service providers in health and social care settings, particularly those who regularly work with people who use drugs should work towards the implementation of the recommendations outlined in this paper; this has the potential to decrease service user death and reduce psychological distress associated with complicated bereavements.

### Recommendations for Policy and Practice

Policies and procedures in addiction must recognize and respond to drug-related complicated grief outcomes as significant public health risks, and it is crucial that responses employ case-sensitive and evidence-based models of practice. The concept of a double stigma acts as an under-acknowledged and hidden harm within the community of people who use drugs, with severe implications for psychological health and mortality. The development of specialized training and education opportunities for staff in social and healthcare settings that cultivate a greater professional understanding of such unique grief experiences would serve to enhance the capabilities of services to address an individualized and complicated grief.

As acknowledged above, given the increasing instances of DRDs, the implementation of therapeutic interventions that integrate both grief and addiction support within service frameworks is crucial. Furthermore, this paper recommends public health campaigns to address the stigma but also raise awareness about the impact of DRDs for the community of people who use drugs. Current research has appealed for the recognition of overdose as a severe psychological trauma (Nolte et al., 2023; Schneider et al., 2021), which finds further credence in the current study as at least one participant disclosed a diagnosis of post-traumatic stress disorder (PTSD) following the DRD of his best friend, but all six participants witnessed fatal and non-fatal overdoses while in active addiction.

### Strengths, Limitations, and Directions for Future Research

From the individual perspectives of six drug death bereaved individuals, this study presents further evidence that people in active addiction grieve for the loss of their peers within their community. However, recruitment from this project took place in an area where all participants' experiences reflect active addiction, grief, and recovery within urban settings. Literature suggests that people who use drugs in rural areas are often exposed to more isolation, so future research should aim to understand the experiences of those who may not be routinely connected to communal spaces. Furthermore, there are avenues to explore cultural differences, such as the experiences of ethnic minority groups.

### Conclusion

The findings of this paper contribute to the understanding of disenfranchised grief within a community that is already marginalized due to systemic stigmas associated with addiction. Individual grief correlates emerge based on group characteristics, but also from distinct within-group individualized factors such as connection to the deceased, allostatic load, and social positioning. The findings contribute to a broader understanding of the social context of drug-related bereavement, and how the intricacies of social positioning can exacerbate grief outcomes in different ways. As a critically under-researched area of inquiry, understanding variability in experience and support needs is crucial to the development of functional and adaptive practice frameworks that address specific psychological harms.
